# Comprehensive Characterization of Alternative mRNA Splicing Events in Glioblastoma: Implications for Prognosis, Molecular Subtypes, and Immune Microenvironment Remodeling

**DOI:** 10.3389/fonc.2020.555632

**Published:** 2021-01-26

**Authors:** Liang Zhao, Jiayue Zhang, Zhiyuan Liu, Yu Wang, Shurui Xuan, Peng Zhao

**Affiliations:** ^1^ Department of Neurosurgery, The First Affiliated Hospital of Nanjing Medical University, Nanjing, China; ^2^ Department of Respiratory and Critical Care Medicine, The First Affiliated Hospital of Nanjing Medical University, Nanjing, China

**Keywords:** glioblastoma, alternative splicing, prognostic signature, molecular classification, immune microenvironment

## Abstract

Alternative splicing (AS) of pre-mRNA has been widely reported to be associated with the progression of malignant tumors. However, a systematic investigation into the prognostic value of AS events in glioblastoma (GBM) is urgently required. The gene expression profile and matched AS events data of GBM patients were obtained from The Cancer Genome Atlas Project (TCGA) and TCGA SpliceSeq database, respectively. 775 AS events were identified as prognostic factors using univariate Cox regression analysis. The least absolute shrinkage and selection operator (LASSO) cox model was performed to narrow down candidate AS events, and a risk score model based on several AS events were developed subsequently. The risk score-based signature was proved as an efficient predictor of overall survival and was closely related to the tumor purity and immunosuppression in GBM. Combined similarity network fusion and consensus clustering (SNF-CC) analysis revealed two distinct GBM subtypes based on the prognostic AS events, and the associations between this novel molecular classification and clinicopathological factors, immune cell infiltration, as well as immunogenic features were further explored. We also constructed a regulatory network to depict the potential mechanisms that how prognostic splicing factors (SFs) regulate splicing patterns in GBM. Finally, a nomogram incorporating AS events signature and other clinical-relevant covariates was built for clinical application. This comprehensive analysis highlights the potential implications for predicting prognosis and clinical management in GBM.

## Introduction

Glioblastoma (GBM) is the most common primary brain neoplasm in adults (1). Although given surgical resection followed by radiotherapy and temozolomide chemotherapy, GBM patients still show poor prognosis with a median overall survival of fewer than 15 months (2). One of the reasons accounting for the limited efficacy of regular treatment is the incomplete debulking surgery, which is subjected to the penetration of malignant cells into healthy tissues. Resistance to conventional chemotherapeutic drugs also poses a critical challenge to clinical GBM therapy (3). Therefore, an improved understanding of the molecular mechanisms underlying GBM progression for developing accurate prognostic markers and novel effective therapeutic strategies is urgently required.

The surge of advances in next-generation sequencing and other multi-omics technologies have made the revelation of the genomic features of GBM increasingly feasible (4, 5). Various pathways involved in gliomagenesis have been widely studied and improved our understanding of the complex mechanisms of GBM, such as *PI3K*/*AKT*/*mTOR*, *PDGF*/*PDGFR*, receptor tyrosine kinase, and *p53* signaling. More importantly, high heterogeneity is a hallmark of GBM, and different GBM molecular subclasses were categorized based on gene expression or methylation data, which may guide the diagnosis and clinical therapy (6–9). However, these molecular classifications have not considered alternative splicing (AS) events.

AS has been proved to be a key factor underlying functional complexity in eukaryotes cells (10). Splicing of pre-mRNA contributes to proteomic diversity from a limited number of genes and therefore regulates the vast majority of biological processes and cellular phenotypes, some of which could be associated with malignant progression (11, 12). AS has been reported to be a universal phenomenon during regular cell activities, with approximately 95% of genes underwent this process (13). Recently, the AS landscapes in several human cancer types have been delineated, including head and neck squamous cell carcinoma, pancreatic ductal adenocarcinoma, colorectal cancer, and hepatocarcinoma (14–17). Cancer-related AS events can not only serve as predictive or prognostic factors but also as effective therapeutic targets. For example, Hu et al. found that MET-exon-14-skipping (*METex14*) was significantly enriched in secondary GBMs (sGBMs) and was responsible for the bleak prognosis. A MET kinase inhibitor targeting this specific splicing pattern has been invented and applied to the clinical treatment of sGBM patients (18). Also, accumulating evidence shows that the dysregulation of AS in the tumor can influence and remodel the microenvironment of the neoplastic niche (19, 20). In GBM, malignant niche recruits immune cells to create an immunosuppressive tumor microenvironment, therefore facilitate the proliferation and encroachment of tumor cells into normal tissues (21). To date, there have been very few systematic studies conducted to investigate the cross-talk between AS events and cancer-immune in GBM.

In our study, we deeply mined the AS events in GBM based on the large-scale transcriptome data from The Cancer Genome Atlas Project (TCGA). Overall survival-related AS events were identified, and an AS events-based signature was constructed to predict the clinical outcomes of GBM patients. Integrative bioinformatics analyses were carried out to explore the underlying biological mechanisms contributed by AS events. Additionally, we classified GBM into two distinct subtypes, and further assessed the association between these clusters and prognoses, clinicopathological features as well as the immune microenvironment. Finally, we developed a robust prognostic model to direct the decision-making in clinical management. Our study revealed the AS landscape of GBM and may shed new light on developing novel therapeutic methods.

## Materials and Methods

### Data Acquisition and Pre-Processing

In this cohort study, RNA sequencing data were obtained from the TCGA patients diagnosed with glioblastoma. Publically available level 3 transcriptome fragments per kilobase million (FPKM) data of the TCGA-GBM project (n = 166, Illumina HiSeq platform) were downloaded from the TCGA data portal (https://portal.gdc.cancer.gov). In the present study, FPKM values were transformed into transcripts per kilobase million (TPM) values, which are more comparable between different samples (22). Raw counts data of these corresponding GBM samples were also downloaded for differential expression analysis. The GISTIC copy number and methylation status of splicing factors in the TCGA-GBM cohort were downloaded from cBioPortal for Cancer Genomics (https://www.cbioportal.org/). Curated clinical information of these glioblastoma patients was derived from a TCGA Pan-Cancer research (23).

AS events profile of 152 glioblastoma patients in the selected TCGA-GBM cohort, a matrix consists of the Percent Spliced In (PSI) value of each sample, was obtained from the TCGA SpliceSeq database (https://bioinformatics.mdanderson.org/TCGASpliceSeq/PSIdownload.jsp). PSI value means the ratio between exons inclusion and exclusion read counts and indicates the efficiency of certain splicing process (24). To ensure the reliability of subsequent analyses, we predefined stringent filtering criteria: the PSI values of AS events in all GBM samples with a standard deviation > 0.05, mean > 0.1, and the percentage of samples with PSI values > 80 were included.

### Identification of Prognostic AS Events and Construction of an AS Events-Based Risk Model

Univariate Cox regression analysis was carried out to find out the prognostic relationship between AS events and overall survival of GBM patients. AS events with P-value less than 0.05 in the univariate Cox regression analysis were regarded as survival-related factors and were selected for the next analysis. The least absolute shrinkage and selection operator (LASSO) cox model using 10-fold cross-validation was performed on the top 20 significant survival-related AS events that have the highest prognostic value to select the most useful prognostic markers of all types of AS events. The optimal lambda value was estimated based on the minimum criteria. Then, these candidate survival-related AS events were further subjected to multivariate Cox regression analysis (25). The risk-score model of AS events for predicting survival of GBM was constructed based on the combination of an AS PSI value and the corresponding regression coefficient (β) obtained from the multivariate Cox regression analysis (26). The formula of calculating the risk score of each GBM patient was as follows:]

$$\text{Risk score}=\sum_{i=1}^{N}(PSI_{ASi}\times \beta_{ASi})$$

where N is the number of AS events in the signature, β_ASi_ is the regression coefficient, and PSI_ASi_ represents the PSI value of a certain AS event in a single sample. GBM patients were divided into a high- and low-risk group according to the median risk score. Kaplan-Meier survival analysis based on the log-rank test was used to evaluate the predictive value of the risk model. Hazard ratios (HRs) and 95% confidence intervals (95% CIs) were calculated both in univariate and multivariate Cox regression analyses. The following clinical-relevant factors were included in the multivariate Cox regression analysis: gender, age, *MGMT* methylation status, Karnofsky Performance Status (KPS) score, *IDH* mutation status, and risk score. Finally, time-dependent receiver operating characteristic (ROC) curves were performed using the “timeROC” R package (v0.4) to display the accuracy of this risk model in predicting the clinical outcomes of GBM patients at different times.

### Similar Network Fusion and Clustering

Based on all survival-related AS events, a modified clustering method, SNF-CC, which combining Similar network fusion (SNF) (27) with Consensus clustering (CC) (28), was applied to perform classification of all GBM patients in the cohort. SNF-CC algorithm was executed by “ExecuteSNF.CC” function implanted in “CancerSubtypes” R package (v1.12.1) (29). To guarantee a balance between high stability and low ambiguity, the detailed parameters were set as follows: clusterNum = 2, K = 20, alpha = 0.5, t = 20, maxK = 5, pItem = 0.8 and reps = 500. The consensus heatmap and cumulative distribution function (CDF) were used to select a more appropriate number of clusters. Furthermore, silhouette width, an index represents how similar a sample is matched to its identified cluster compared to other clusters, was validated in our study. The associations between AS events-based subtypes, clinicopathological features (age, overall survival status, KPS, *IDH* mutation, and *MGMT* methylation status), GBM molecular subtypes (Verhaak classification and EM/PM classification), and immune features (described below) were evaluated.

### Functional Enrichment Analyses

Gene set enrichment analysis (GSEA) analysis was conducted using the R package “fgsea” (v1.12.0) (30). We ranked the GBM samples according to their log2-fold change value (from high to low) derived from differential expression analysis between two distinct groups and checked if any signaling pathway or molecular hallmark was associated with these differentially expressed genes. GSEA gene sets (curated gene sets (C2), Gene Ontology (GO) gene sets (C5), and hallmark gene sets (H)) were downloaded from the Molecular Signatures Database (v7.0) (https://www.gsea-msigdb.org/gsea/msigdb/index.jsp). To obtain robust results, gene-set permutations were performed 10,000 times, and enrichment P-values were adjusted by false discovery rates (FDR). FDR-adjusted P < 0.05 were considered as statistically significant. GO term enrichment and the Kyoto Encyclopedia of Genes and Genomes (KEGG) pathway analysis for genes of interest were performed using metascape (v3.5), which is an easy-to-use web portal that provides a comprehensive analysis for the functional annotation of lists of genes (31).

### Single-Sample Gene Set Enrichment Analysis (ssGSEA)

The fractions of diverse infiltrated immune cells in tumor samples were estimated using the ssGSEA method implemented in the “GSVA” R package (v1.34.0, https://bioconductor.org/packages/release/bioc/html/GSVA.html). As an extension of Gene Set Enrichment Analysis (GSEA), ssGSEA evaluate the enrichment score of a certain gene set in every single sample (32). Twenty-four kinds of immune cell marker genes derived from a previously published research were integrated into immune cells specific gene sets (33). Markers associated with cells of the innate immune system, including natural killer (NK) cells, NK CD56dim cells, NK CD56bright cells, dendritic cells (DCs), immature DCs (iDCs), activated DCs (aDCs), neutrophils, mast cells, eosinophils, and macrophages, as well as those associated with cells of the adaptive immune system, including B, T central memory (Tcm), CD8+ T, T effector memory (Tem), T follicular helper (Tfh), Tγδ, Th1, Th2, Th17, and Treg cells, were included in the gene sets list. Finally, ssGSEA captured a numeric matrix containing enrichment scores of different immune cells across all GBM samples.

### Evaluation of Immune Features

Immune infiltration features in GBM samples can be inferred by calculating tumor immunological indexes. According to a pan-cancer study aiming at developing an effective biomarker for predicting immunotherapy response (34), immune infiltration score (IIS), T cell infiltration score (TIS), antigen presentation machinery score (APS), and tumor immunogenicity score (TIGS) were computed. Briefly, IIS indicated total immune infiltration level in the tumor sample and was calculated as the mean of standardized infiltration scores for all kinds of immune cells obtained from the GSVA algorithm. Similarly, TIS was calculated using T cell subsets, including CD8+ T, T helper, T, Tcm, Tem, Th1, Th2, Th17, and Treg cells. APS was calculated with GSVA using APM related genes (*PSMB5*, *PSMB6*, *PSMB7*, *PSMB8*, *PSMB9*, *PSMB10*, *TAP1*, *TAP2*, *ERAP1*, *ERAP2*, *CANX*, *CALR*, *PDIA3*, *TAPBP*, *B2M*, *HLA-A*, *HLA-B*, and *HLA-C*). Tumor burden (TMB) was the number of non-synonymous mutations per megabase (MB). Here, we used 38 MB as the estimate of exome size and defined the TMB as the quotient of non-synonymous mutations and 38MB. Then, TIGS was calculated by multiply APS and TMB value. Predicted abundance of neoantigens in GBM samples was obtained from a previous study (35).

### Prediction of Responses to Immune Checkpoint Blockade (ICB) Therapy

The subclass mapping method (SubMap) (36) was used to compare the gene expression matrices of different GBM subtypes with the expression profiles of several cancer types treated with ICB therapies, including transcriptomic data of 47 melanoma patients who received immunotherapy targeting *CTLA-4* and *PD-1*, and NanoString data of 49 patients with four cancer types treated with anti-PD1 therapy (37, 38). This step was performed using the SubMap module (v3) on the GenePattern website (http://genepattern.broadinstitute.org/). Parameters of SubMap analysis were set as default: num marker genes = 100, num perm = 100, and num perm fisher = 1,000. P-values were adjusted by Bonferroni correction.

### Splicing Correlation Network Construction

A list of splicing factors (SFs) was obtained from the SpliceAid2 database (http://www.introni.it/splicing.html). SpliceAid2 database embodies experimental curated splicing factors to help researchers understand the tissue-specific alternative splicing. The SF gene expression profile of GBM patients was retrieved from TCGA RNA-seq data. Overall survival-related SFs were determined using univariate Cox regression analysis. The correlation of SFs and AS events was evaluated by Pearson correlation analysis, and the regulatory network plot was generated in Cytoscape software (v3.8.0).

### Construction and Validation of the Nomogram

Multivariable Cox proportional hazards regression analysis was applied with the following clinical-relevant covariates: gender, age, *MGMT* methylation status, Karnofsky Performance Status (KPS) score, *IDH* mutation status, and risk score. A combined nomogram was generated by the “regplot” R package (v1.0) as a quantitative tool for predicting the likelihood to die of each patient. The concordance index (C-index) was calculated to assess the consistency between model prediction and actual clinical outcomes of patients. The calibration plot was generated to evaluate the accuracy of the prediction for 1- and 2- year overall survival using this nomogram by the “rms” R package (v5.1-4). Additionally, decision curve analysis (DCA) was applied to evaluate the performance of the nomogram by the “rmda” R package (v1.6).

### Statistical Analysis

The intersections of seven types of AS events in GBM were plotted using the “UpSetR” R package (v1.4.0) (39). The “glmnet” package (v3.0-2) was used to conduct the LASSO Cox regression model analysis. Kaplan-Meier survival curves were visualized to discover the difference of clinical outcomes between groups using “survival” and “survminer” R package (v0.4.6). 3D-PCA plot was generated using “mixOmics” (6.10.9) (40) and “rgl” (v0.100.5) (https://cran.r-project.org/web/packages/rgl/index.html) R package.

Restricted mean survival (RMS) represents the loss in average life expectancy for patients. We performed RMS time calculation based on the univariable Cox proportional hazards regression of overall survival (41). RMS and time ratio were estimated using the “survRM2” R package (v1.0-2).

ESTIMATE is a widely used method that can infer the fraction of immune and stromal cells in tumor samples using the gene expression profile (42). Tumor purity was defined as the percentage of malignant cells in a solid tumor sample. Here, the purity of each GBM sample was assessed by package “ESTIMATE” (v1.0.13, https://bioinformatics.mdanderson.org/estimate/rpackage.html). Furthermore, the tumor purity was further validated using the “TPES” R package (v1.0.0), which is a computational method for estimating tumor purity from single-nucleotide variants. Here, the tumor purity indexes of the TCGA-GBM samples calculated using the “TPES” algorithm were derived from the supplementary files of published literature (43).

All statistical analyses were performed using R (version 3.6.1, www.r-project.org), with chi-square or Fisher’s exact test for patients’ characteristics in different clusters, a Mann-Whitney U test for testing the differences in means of continuous data, and log-rank test for survival analysis. For all hypothetical tests, a two-sided P-value < 0.05 was considered to be statistically significant.

## Results

### The Landscape of AS Events in GBM

**Figure 1:**
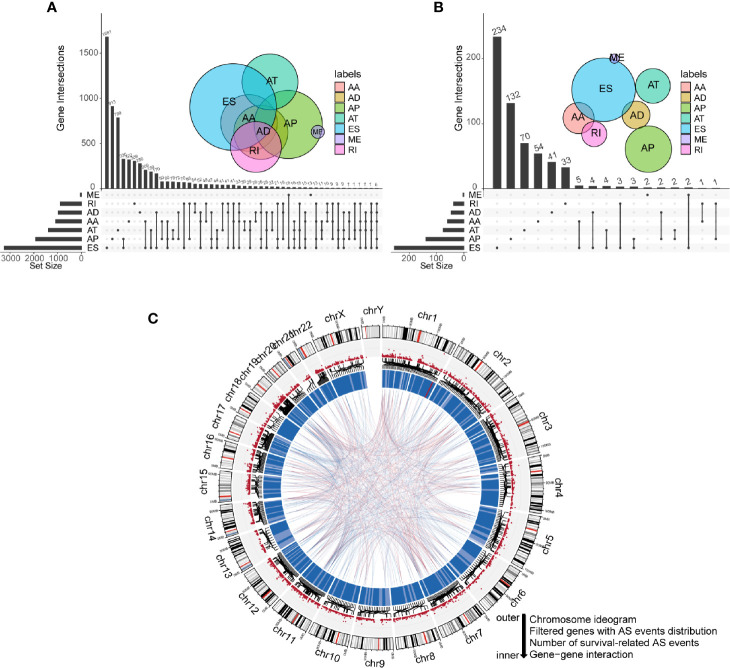
Profiling of all and prognostic alternative splicing (AS) events in glioblastoma (GBM). **(A)** UpSet diagram of interactions between all alternative splicing events in GBM. **(B)** UpSet diagram of interactions between overall survival-related AS events in GBM. **(C)** Circos plot of all AS events, prognostic AS events, and their parent genes in chromosomes. The circle panels from the outer to inner were described as follows: chromosome ideogram, genes with AS events distribution, number of survival-related AS events, and gene-gene interaction.

Integrated mRNA AS events profile was analyzed for 152 GBM patients from the TCGA cohort. Under the stringent filtering criteria, a total of 15,907 AS events from 6,491 genes were identified (**Table S1**). These AS events consisted of seven different splicing patterns: Alternate Acceptor site (AA), Alternate Donor site (AD), Alternate Promoter (AP), Alternate Terminator (AT), Exon Skip (ES), Retained Intron (RI), and Mutually Exclusive Exons (ME). Visualization of specific numbers and intersections of all types of AS events in GBM was generated in **Figure 1A**. Among all these types, ES and AP splicing modes accounted for almost 60% of the total number (9,478/15,907). Notably, a single gene may have more than one type of splicing patterns. We detected 2,184 genes contain two or more types of AS events in GBM samples. For instance, *PDE4DIP* has six types of AS events, including AA, AD, AP, AT, ES, and RI.

To explore the correlations between AS events and the overall survival of GBM patients, we performed univariate Cox regression analysis using PSI values of all AS events and survival information of patients. Eventually, 775 AS events of 593 genes were confirmed to be prognostic in GBM (*P-*value < 0.05). Among them, we noticed that some genes have more than one type of prognostic AS events. For example, AA and ES splicing modes of *ATG4D* were all associated with overall survival. The UpSet plot diagram was illustrated to visualize the interactions among these AS events (**Figure 1B**). A more intuitive landscape of AS events and their prognostic value in GBM was shown using the circos plot (**Figure 1C**).

### Construction of an AS Events-Based Prognostic Model for GBM

**Figure 2:**
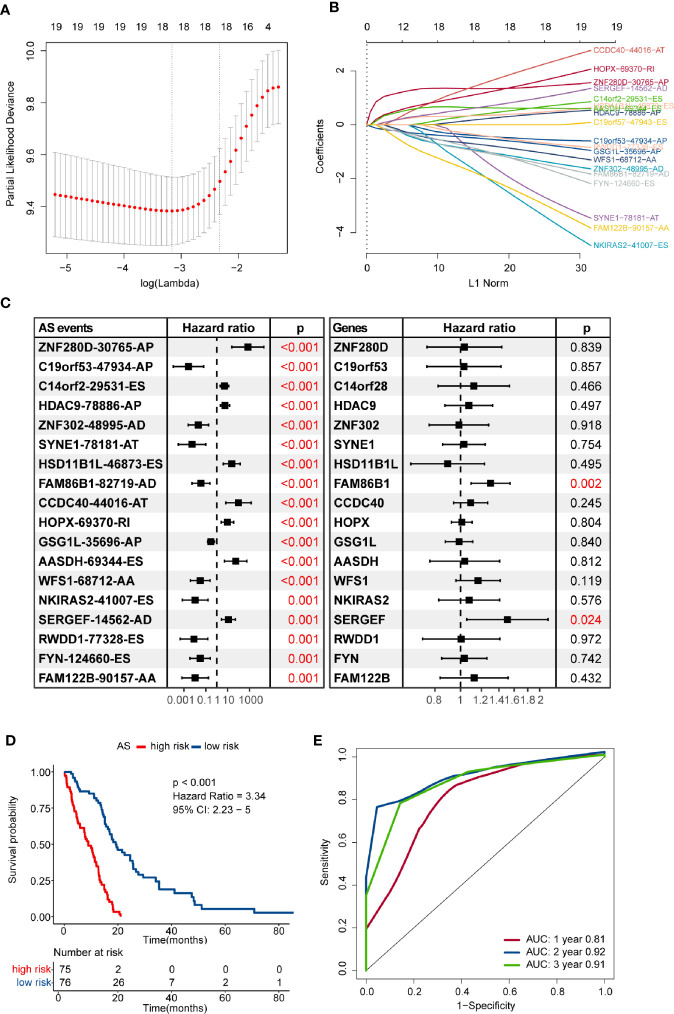
Construction and evaluation of an alternative splicing (AS) events-based risk model. **(A)** The partial likelihood deviance was plotted using vertical lines with red dots, and the dotted vertical lines represent values based on minimum criteria and 1-SE criteria, respectively. A value λ = 0.035 with log(λ) = -3.349 was chosen *via* minimum criteria. **(B)** Least absolute shrinkage and selection operator (LASSO) coefficient profiles of the candidate AS events by 10-fold cross-validation. **(C)** Prognostic value of the candidate AS events and their corresponding genes in the glioblastoma (GBM) cohort. The hazard ratio (HR) and P-values were calculated using the univariate Cox regression analysis. **(D)** Comparison of overall survival according to the AS events-based signature for patients. **(E)** Time-dependent receiver operating characteristic (ROC) analysis was used to assess the performance of the risk model in predicting 1-, 2- and 3-year survival of GBM patients.

Numerous survival-related AS events were identified after performing univariate Cox regression analysis. In order to get the AS events with the greatest potential prognostic value, LASSO Cox regression analysis was carried out, and 18 AS events were finally selected (**Figures 2A, B**). We also validated whether the original genes of these 18 AS events are survival-related factors when applying the univariate Cox regression analysis to the corresponding gene expression profiles, and we found that only two of them remain statistically significant, including *SERGEF* and *FAM86B1* (*P-*value < 0.05) (**Figure 2C**). Besides, only *SERGEF* and its splicing isoform, *SERGEF-14562-AD*, shared similar prognostic value, while *FAM86B1* and *FAM86B1-82719-AD* had the reverse patterns in prognostic value. As revealed above, we identified and characterized that isoform-based analysis can capture some meaningful transcripts that were not available for gene-level analysis.

Then, we developed a formula to compute the risk score for each GBM patient based on the PSI values and regression coefficients of AS events. The risk score of each GBM sample ranged from -13.782 to -7.970, and the median score of -10.569 was defined as the cut-off to separate the whole GBM cohort into high- and low-risk groups. The efficacy of the risk model for predicting overall survival of GBM patients was evaluated by performing Kaplan–Meier survival analysis. The result indicated that the risk score-based signature was significantly associated with the prognosis of GBM patients. Patients in the high-risk group had worse clinical outcomes than those in the low-risk group (HR = 3.34, 95% CI: 2.23–5, P < 0.001) (**Figure 2D**). The performance of the risk model was assessed in terms of the RMS time ratio between different risk groups, and significant shorter RMS time were observed in high-risk group compared with their low-risk counterparts (RMS_low-risk_/RMS_high-risk_ = 1.793, 95% CI: 1.514–2.123, P < 0.001) (**Table S2**). Time-dependent ROC curves evaluated the performance of this risk model in predicting outcomes, and the area under the ROC curve was 0.81 at 1-year, 0.92 at 2-year, and 0.91 at 3-year, respectively (**Figure 2E**).

**Figure 3:**
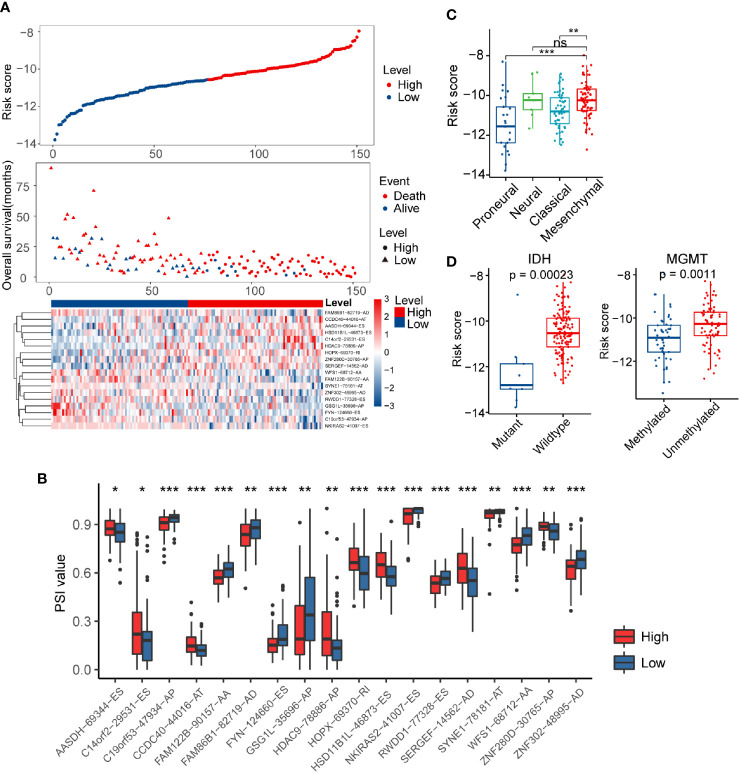
Distribution of the risk score and differences in clinicopathological features in glioblastoma (GBM) patients. **(A)** Overview of risk score, clinical outcomes of patients, and expression heatmap of 18 alternative splicing (AS) events in the signature. **(B)** Boxplots visualizing the different levels of Percent Spliced In (PSI) values between the high- and low-risk groups. **(C)** Risk score in different molecular subtypes of The Cancer Genome Atlas Project (TCGA) classification scheme. **(D)** Comparison of risk score according to *IDH* mutation status or *MGMT* methylation status. * indicates *P* < 0.05; ** indicates *P* < 0.01; *** indicates *P* < 0.001.

The risk score distribution, survival status, and AS events profile of this signature were shown in **Figure 3A**. More patients were found dead in the high-risk group than the low-risk group, and the overall survival time of high-risk patients was much shorter than patients of low-risk. We further calculated the different usage of the transcript isoforms in the alternative splicing signature in the different risk groups. The PSI value represents the frequency of the specific transcript isoform that occurred in the alternative splicing process. Therefore, we compared the PSI values of these AS events in the risk model signature to reflect the differential usage of the mRNA isoforms. The abundance of the risky AS events with HR greater than one in the high-risk group were more than the low-risk group, while the opposite pattern occurred in the protective AS events (**Figure 3B**). These results indicated that the substantial differences in survival between high- and low-risk groups might result from the isoform switching.

Verhaak’s GBM molecular classification was developed based on the gene expression profile of the TCGA GBM cohort and has been widely used for GBM research (9). The Verhaak subtype information of each sample in the present study was obtained from UCSC Xena (https://xenabrowser.net/). Next, we investigated the inter-tumor heterogeneity of risk score by examining the relationship of risk score with different Verhaak’s subtypes. Higher scores were found in the mesenchymal subtype compared to the classical and proneural subtype (**Figure 3C**). *IDH* mutation and *MGMT* methylation status are well-documented GBM molecular markers that can predict the overall survival of patients (44). In general, *IDH* wild-type and *MGMT* unmethylated status usually indicate a worse prognosis. We separated GBM patients into different groups based on these two biomarkers to explore the association between risk score and *IDH* mutation/*MGMT* methylation status (**Figure 3D**). We noticed that the risk score was significantly higher in the *IDH* wild-type group than the *IDH* mutation group (*P* < 0.001). A similar result was also found in *MGMT* unmethylated group (*P* < 0.01). Furthermore, the HRs for the AS events-based signature in the univariate and multivariate Cox regression analyses were 2.718 (*P* < 0.001, 95% CI: 2.186–3.38) and 2.496 (*P* < 0.001, 95% CI: 1.802–3.458), respectively (**Table S3**). Thus, the risk score model was proved to be an independent prognostic factor for GBM patients.

### SNF-CC Identified Two Distinct Subtypes of GBM Patients

**Figure 4:**
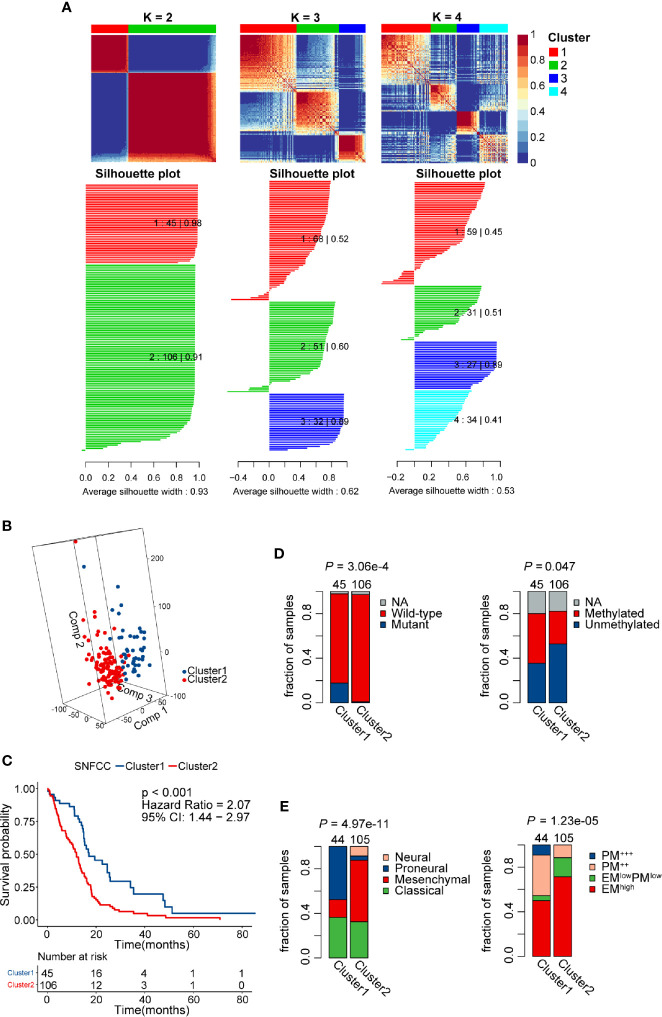
Identification of two distinct clusters using the similarity network fusion and consensus clustering (SNF-CC) method. **(A)** Heatmap of the sample similarity matrix and silhouette width plots of the subtypes for k = 2 to 4. **(B)** 3D-PCA (Principal Components Analysis) plot of patients in different clusters. Comp 1, Comp 2, and Comp 3 on axes represent three principal components respectively. **(C)** Comparison of overall survival for patients of different clusters. **(D)** Novel glioblastoma (GBM) classification was associated with *IDH* mutation and *MGMT* methylation status. Cluster2 tumors had a significantly higher *IDH* wild-type and *MGMT* unmethylation rate. **(E)** Significant associations between alternative splicing (AS) events-based classification and other GBM molecular subtypes. Cluster2 tumors had a significantly higher mesenchymal and EM subtype rate.

Patients without detailed survival information were excluded before performing the SNF-CC algorithm, and then PSI values of AS events in all samples were scaled *via* z-score standardization. The built-in Cox model function of the “CancerSubtypes” package enabled us to filter crucial survival-related splicing patterns specific to GBM. The performance of this clustering method was assessed by clustering heatmap, CDF curves, and silhouette width. The results demonstrated that SNF-CC achieved adequate robustness when all GBM patients were categorized into two distinct clusters, with Cluster1 consists of 45 patients and Cluster2 of 106 patients (**Figure 4A**, **Figure S1**). PCA analysis supported the effectiveness of this clustering method in defining GBM sub-grouping based on survival-related AS events (**Figure 4B**). Importantly, statistically significant differences in outcomes of patients were observed in these two clusters, and Cluster2 patients showed worse prognoses than those of Cluster 1 (HR = 2.07, 95% CI: 1.44–2.97, *P* < 0.001) (**Figure 4C**). A higher proportion of patients with *IDH* wild-type (fractions, 99.03% vs 81.82%, P = 3.06e-4) and *MGMT* unmethylation (fractions, 64.37% vs 44.44%, P = 0.047) was observed in Cluster2 compared with Cluster1 (**Figure 4D**). Also, we found a significant difference in Verhaak’s subtype among two clusters (P = 4.97e-11), and Cluster2 had more mesenchymal GBMs than Cluster1 (fractions, 55.24% vs. 15.91%) (**Figure 4E**). The EM/PM subtype is another glioma molecular classification based on the coexpression modules of *EGFR* and *PDGFRA* (45). The prognosis of EM glioma was much worse than PM glioma. Here, we noticed a significant relationship between EM/PM subtype and the novel classification scheme we developed (P = 1.23e-5), and Cluster2 had more EM^high^ samples than Cluster1 (fractions, 71.43% vs 50%).

**Figure 5:**
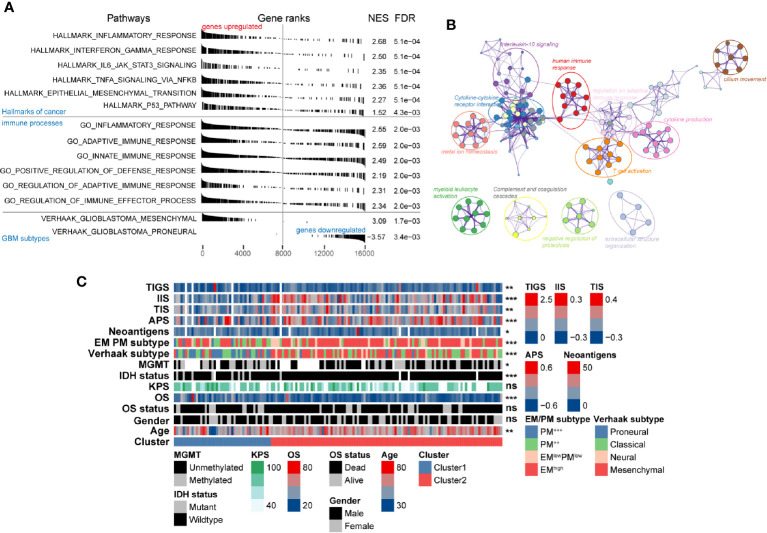
Functional annotation of the molecular differences and comparison of immunological features in different clusters. **(A)** Gene set enrichment analysis showing significant enrichment of various signaling pathways and gene sets in Cluster2 compared with that in Cluster1. The label of “genes upregulated” and “genes downregulated” represent genes upregulated/downregulated in Cluster2 compared with Cluster1. **(B)** Network of biological processes and signaling pathways enriched in genes upregulated in Cluster2 compared with Cluster1. **(C)** The association between novel alternative splicing (AS)-based glioblastoma (GBM) classification, clinicopathological factors, GBM subtypes, and immune features was annotated in the heatmap.

The remarkable differences in both clinicopathological and molecular characteristics between these two clusters suggested that different functional annotations and signaling pathways might exist. GSEA manifested a remarkable activation in cancer-associated signaling pathways was significantly enriched in Cluster2 compared with Cluster1, including epithelial-mesenchymal transition (NES = 2.27, FDR = 5.1e-4), P53 (NES = 1.52, FDR = 4.3e-3), *IL6 JAK STAT3* signaling (NES = 2.35, FDR = 5.1e-4), and interferon-gamma response pathways (NES = 2.5, FDR = 5.1e-4) (**Figure 5A**, **Table S4**). Besides the cancer hallmark biological processes, Cluster2 was also involved in various immune-related responses, such as inflammatory response (NES = 2.55, FDR = 2e-3), adaptive innate immune response (NES = 2.59, FDR = 2e-3), innate immune response (NES = 2.49, FDR = 4.3e-3), and immune effector process regulation (NES = 2.34, FDR = 2e-3). Overexpressed genes in Cluster2 (log2-fold change > 1 and adjusted P-value < 0.05) were calculated as input for functional annotation analyses using Metascape. The GO and KEGG enrichment analysis indicated that Cluster2 had enriched genomic biological processes, including human immune response, cytokine-cytokine receptor interaction, T cell activation, cytokine production, *IL-10* signaling, and regulation of leukocyte migration (**Figure 5B**).

### Association of Identified Subtypes With Immune Cell Infiltration in the GBM Microenvironment

The intratumoral microenvironment is a complex that consists of the tumor and non-tumor cells, such as stromal and immune cells (46). These non-malignant cells can regulate the progression of tumorigenesis *via* the cross-talk with malignant cells in GBM (47). We found that both IIS and TIS were much higher in Cluster2 than in Cluster1, which represented higher relative fractions of total immune cells (P < 0.001), and T cell subsets (P < 0.01) were infiltrated in tumors of Cluster2 (**Figure 5C**). Recently, immunotherapies targeting immune checkpoints blockade (ICB) had been proved to exhibit critical anti-tumor functions by promoting anti-tumor immune responses and inhibiting immunosuppressive effects. Clinical outcome of ICB therapy has been reported to be closely related to neoantigen abundance in some cancers (48, 49). Here, a significant depletion of the neoantigen burden was found in Cluster2 (P < 0.05). Besides, we also noticed a relatively higher level of APS in Cluster2 compared with Cluster1 (P < 0.001). In glioma, decreasing of neoantigens was tightly associated with intact APM function, increased infiltration of immune cells, and active immune processes (50). This may explain the phenomenon that Cluster2 contained higher APS, IIS, and TIS while harboring lower numbers of neoantigens.

**Figure 6:**
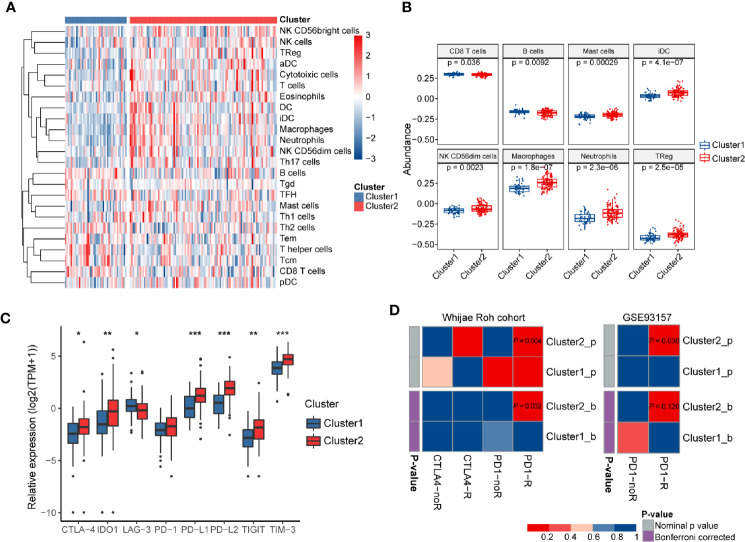
The immune landscapes and predicted immunotherapeutic responses among different clusters. **(A)** The relative proportion of immune cell infiltration in the two clusters obtained by ssGSEA analysis. **(B)** Boxplots visualizing significantly different abundance levels of infiltrated immune cells between the two clusters. **(C)** Comparison of the mRNA expression levels of several different immune checkpoints between these two clusters. **(D)** SubMap analysis of the GBM subtypes and two independent immunotherapeutic treatment datasets. SubMap analysis suggested that patients of Cluster2 may be more sensitive to anti-PD-1 immunotherapy. The colors in the cells represent the nominal and Bonferroni corrected p values.

To further validate the association between immune system processes and these two subtypes, the ssGSEA method was performed to estimate the differences in the detailed immune infiltration of 24 types of immune cells between these clusters (**Figure 6A**). We found that Cluster2 exhibited a higher abundance of pro-tumor immune cell types, including mast cells, immature dendritic cells (iDCs), CD56^dim^ natural killer cells, macrophages, neutrophils, and regulatory T cells (Tregs) (**Figure 6B**). Meanwhile, anti-tumor immune cells, such as CD8+ T cells and B cells, were significantly significantly depleted in Cluster2 samples. These results indicated that the enrichment of numerous immune-related pathways in Cluster2 might result from the differences in recruitment or differentiation of diverse immune cells in tumors.

Wang et al. pointed out that TIGS is an ideal index to predict clinical response to ICB therapy (33). Therefore, a higher TIGS level in Cluster2 suggested more patients may benefit from ICB therapy than those of Cluster1. The expression levels of immune checkpoints have been proposed to be useful references for selecting patients receiving immunotherapy. Thus, we further investigated the expression levels of several well-known immune checkpoints (*TIM-3*, *TIGIT*, *LAG-3*, *PD-1*, *CTLA-4*, *PD-L1*, *PD-L2*, and *IDO1*) between two distinct clusters. Most of them were more highly expressed in Cluster2 than in Cluster1 (P < 0.05) (**Figure 6C**). Finally, SubMap analysis manifested that samples in Cluster2 shared a higher similarity with the expression profile of melanoma patients who were responsive to *PD-1* inhibitor treatment (P = 0.004, Bonferroni P = 0.032) (**Figure 6D**). Moreover, the same procedure performed on another cancer cohort containing 49 baseline tumors in four cancer types, GSE93157, also achieved similar results (P = 0.03, Bonferroni P = 0.12) (**Figure 6D**). These findings confirmed that GBM samples in Cluster2 may benefit from anti-PD-1 therapy compared with those of Cluster1.

### Association of Risk Score and Immunosuppressors

**Figure 7:**
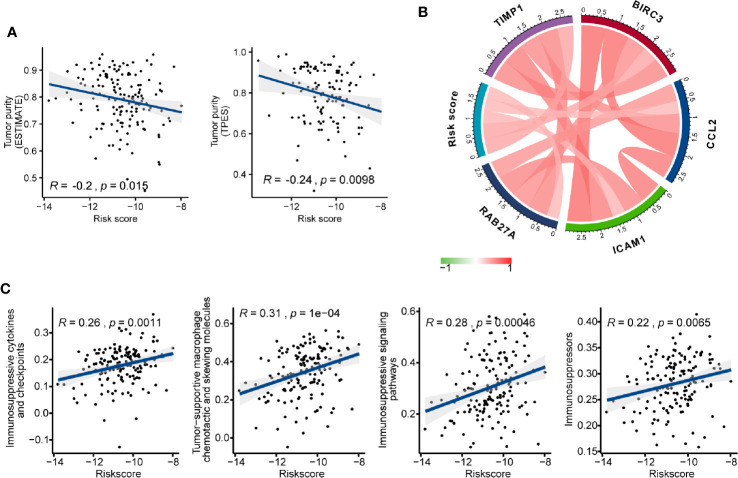
The immunosuppressive function of the alternative splicing (AS) events signature. **(A)** Correlation of risk score with glioblastoma (GBM) purity estimated by the “ESTIMATE” and “TPES” algorithms, respectively. **(B)** Correlation of the risk score with the expression levels of several representative immunosuppressive genes. **(C)** Correlation of risk score with ssGSEA scores of immunosuppressor metagenes.

We investigated the association between AS events-based risk score and tumor purity in GBM using both ESTIMATE and TPES algorithms, and we found a stable significant negative correlation among them (ESTIMATE: Pearson correlation coefficient (R) = -0.2, P = 0.015; TPES: R = -0.24, P = 0.0098) (**Figure 7A**). Meanwhile, higher stromal and immune score were observed in high- than the low-risk group (P < 0.05) (**Figure S2**). We also explored the relationship between risk score and glioma mediated immunosuppression. The gene sets of glioma-related immunosuppressive factors, including immunosuppressive cytokines and checkpoints, tumor-supportive macrophage chemotactic and skewing molecules, immunosuppressive signaling pathways, and immunosuppressors were extracted from previous reports (51, 52). The risk score was positively correlated with the expression of most genes (**Figure S3**). Pearson correlation analysis also found that risk score was positively correlated with immunosuppressive genes including *TIMP1* (R = 0.450), *BIRC3* (R = 0.435), *ICAM1* (R = 0.361), *CCL2* (R = 0.359), and *RAB27A* (R = 0.352) (**Figure 7B**). After that, ssGSEA analysis was carried out to assess the enrichment score of each gene set for every single GBM sample. Positive correlations between risk score and each gene set score were found (**Figure 7C**), indicating that AS events-based signature played a vital role in immunosuppression in GBM microenvironment.

### The Network of Survival-Related AS Events and Splicing Factors

**Figure 8:**
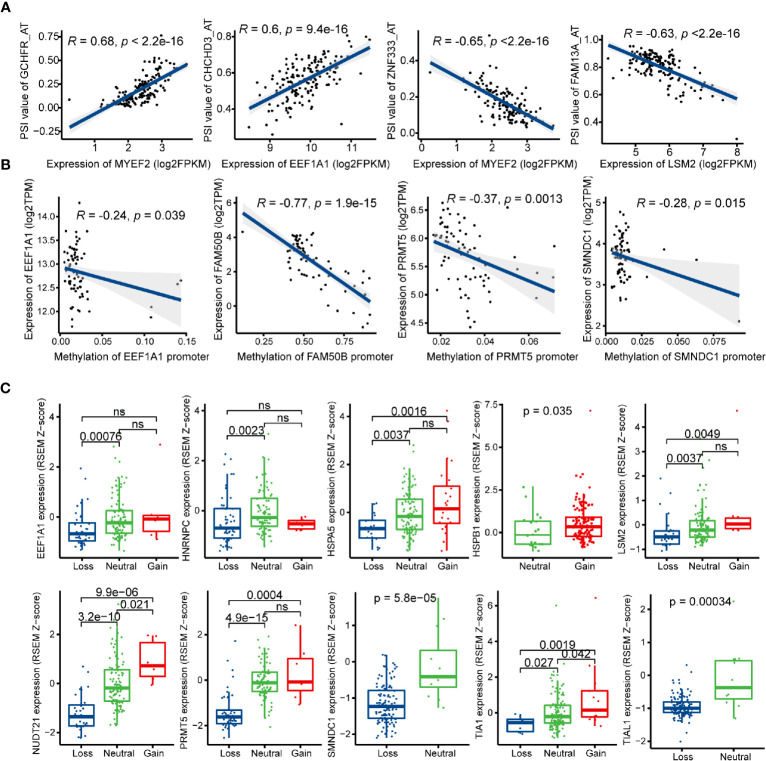
Regulatory mechanisms of splicing factors (SFs) in glioblastoma (GBM). **(A)** Representative correlations between Percent Spliced In (PSI) values of survival-related alternative splicing (AS) events and the expression of SFs. **(B)** Representative correlations between expression of SFs and the SFs promoter methylation. **(C)** Representative boxplots of SFs expression among different copy number status.

SFs play an important role in regulating the process of exons inclusion and introns exclusion during alternatively splicing pre-mRNA. Changes of SFs lead to the production of diverse splicing patterns of genes, including some oncogenic isoforms, and thus promote or inhibit tumorigenesis (53). In order to depict the potential regulatory network between SFs and prognostic AS events, univariate Cox regression analysis was carried out to identify the survival-related SFs in GBM. 16 SFs were regarded as core SFs (P < 0.05), including *CELF6*, *HSPB1*, *HSPA5*, *TIA1*, *PQBP1*, *TIAL1*, *EEF1A1*, *FAM50B*, *NUDT21*, *SMNDC1*, *HNRNPC*, *PRMT5*, *JUP*, *MYEF2*, *LSM2*, and *HNRNPLL*. Significant correlations with |R| > 0.4 and *P* < 0.05 were shown in the network map (**Figure S4**, **Table S5**). A total of 14 prognostic SFs were correlated with 116 AS events. Several most significant correlations were presented in **Figure 8A**.

Posttranscriptional modification of SFs can also influence alternative splicing, such as phosphorylation and methylation (54, 55). In this study, the methylation levels of *EEF1A1*, *FAM50B*, *PRMT5*, and *SMNDC1* promoters were negatively associated with their mRNA expression levels (**Figure 8B**). Next, we investigated the relationships between copy number alteration (CNA) and expression levels of prognostic SFs, and the expression levels of 10/14 SFs were associated with CNA events (**Figure 8C**).

### Development of a Nomogram Based on AS Events

**Figure 9:**
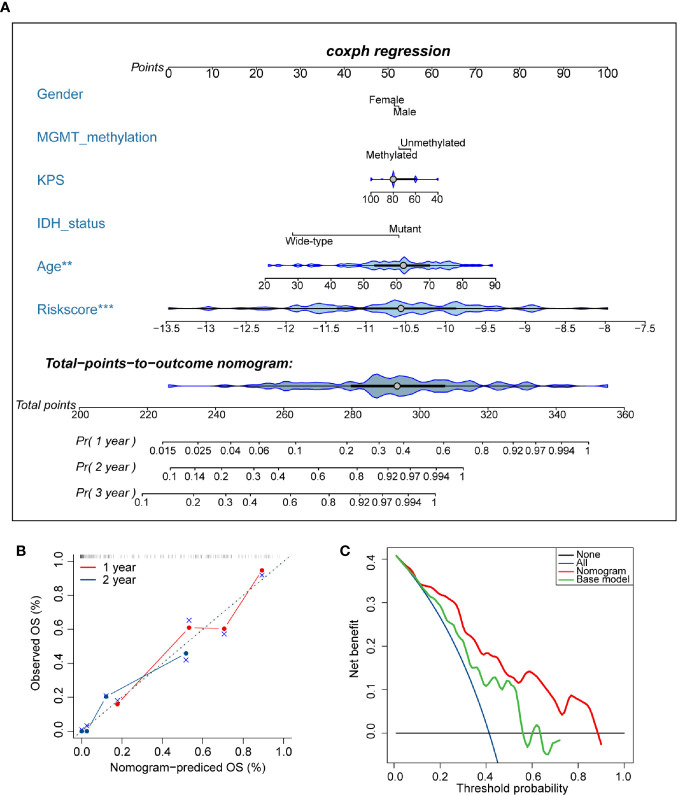
Developed nomogram to predict the risk of death in glioblastoma (GBM) patients. **(A)** Nomogram built with clinicopathological factors incorporated estimating 1-, 2-, and 3-year overall survival for GBM patients. The asterisk beside each variable in the nomogram represents the statistical significance. ** indicates P < 0.01; *** indicates P < 0.001. **(B)** The calibration curves describing the consistency between predicted and observed overall survival at 1- and 2-year. The estimated survival was plotted on the x-axis, and the actual outcome was plotted on the y-axis. The gray 45-degree dotted line represents an ideal calibration mode. **(C)** Decision curve analysis for the nomogram and base model. The red line measures the nomogram, and the green line represents the base model. The selected probability threshold is plotted on the abscissa.

To develop a quantitative tool for predicting the prognosis of GBM patients, we established a nomogram by integrating clinicopathological risk factors and AS events-based signature based on the multivariable Cox proportional hazards model (**Figure 9A**). The point scale in the nomogram was utilized to generate point to these variables, and the risk of death of each GBM patient was qualified by accumulating total points of all variables. The risk score was found to have the most excellent weight among all these variables, which was consistent with the result of the previous multivariable Cox regression analysis. The C-index of this nomogram reached 0.774 (95%CI: 0.743–0.805). The result of the calibration plot further confirmed the significant consistency between predicted and observed actual clinical outcomes of GBM patients (**Figure 9B**). The decision curve analysis showed a higher overall net benefit using the nomogram than either the “treat all” or the “treat none” approach within a range of threshold probabilities > 10% (**Figure 9C**). Moreover, the same result was found when compared with the base model, which contained age, gender, KPS, *IDH* status, and *MGMT* methylation status of patients. All these findings demonstrated that the AS events-based nomogram is an optimal model for predicting the prognosis of GBM patients in clinical management.

## Discussion

Alternative processing of mRNA, a universal phenomenon that happened in the process of transcriptional regulatory, can increase the diversity of protein to a large scale. The advent of next-generation sequencing and the development of bioinformatics make it possible to deeply elucidate the mechanisms of alternative splicing behind various biological processes, such as cancer. Combining with comprehensive multi-omics databases, such as TCGA, the novel role of alternative splicing in human cancers can be further explored. The previous studies had noticed that AS events of specific genes can drive or suppress tumorigenesis of glioma. For instance, *GFAP-δ* and *GFAP-α* are two types of *GFAP* alternative splice variants, and high *GFAP-δ/α* ratio in glioma cells contributes to a more invasive phenotype by activating the expression of *DUSP4* (56). Li et al. demonstrated that β splicing of *hTERT* was tightly linked to higher tumor grades and poor prognosis of glioma patients (57). To our best knowledge, previous researches on AS events of diffuse glioma and GBM mainly focused on limited samples or cancer cell lines, and a large-scaled prognostic AS events in GBM remain largely unstudied.

Compared with microarray assays, RNA-seq is superior in sequencing depth and dynamic range, and more AS events can be identified using RNA-seq data. In recent years, signatures based on gene expression, DNA methylation, and even multi-omics profiles have been widely developed to clarify their clinical relevance in GBM (58–60). Here, transcriptome data of GBM samples in TCGA were obtained, and alternative splicing patterns were calculated using SpliceSeq, which is an effective tool to accurately identify potential AS events using RNA-seq data (61). We established an AS events-based signature as a potential prognostic model to categorize GBM patients into different risk groups. The remarkable discrepancy of overall survival in the two groups suggested the existence of significant tumor heterogeneity. Given that molecular heterogeneity may underlie differences in prognosis and responsivity to clinical therapy, we linked risk score to some GBM molecular features, such as TCGA Verhaak’s GBM classification, *IDH* mutation, and *MGMT* promoter methylation, and we confirmed that these samples are highly variable from patient to patient at a molecular level.

Due to the molecular heterogeneity, the use of the anatomical distribution, WHO grade, *IDH* mutation, and *MGMT* methylation status to classify patients and determinate therapeutic options have limited value. In this study, all survival-related AS events were filtered as input file of SNF-CC clustering, and two AS events-based clusters were identified. Overall survival, *IDH* mutation, *MGMT* methylation, Verhaak’s subtype, and EM/PM subtype were unevenly distributed among these newly identified subtypes. Interestingly, both Verhaak’s and EM/PM classification were based on patterns of gene expression, and the close associations between AS- and gene patterns-based subtype may indicate the resemblance in biological pathways. A series of functional annotation analyses showed that besides classical oncogenic hallmarks, Cluster2 samples also presented a stronger immunophenotype than samples in Cluster1. At first, the brain was regarded as an immunologically privileged organ due to the existence of the blood-brain barrier and the deficit of immune activities. The discovery that the infiltration of multiple immune cell types in CNS has made this view obsolete (62, 63). Thus, it could be speculated that immune signaling pathways exclusively enriched in the Cluster2 subtype might play an important role in driving tumorigenesis. After describing the landscapes of immune cell infiltration among samples of both subtypes, we preliminary noticed that much immune cell types were highly abundant in the Cluster2. However, contrary to what we expected, the prominent immune phenotype contributed to a worse instead of a favorable prognosis because of the large proportions of pro-tumorigenic immune cells as well as less anti-tumorigenic immune cells in these samples. Much more contents of cytotoxic cells, NK cells, neutrophils, and DCs in Cluster2 suggested that these patients may show better responsivities to immunotherapies. Also, as an effective index for ICB-response prediction, TIGS was much higher in Cluster2 compared with Cluster1, indicating more patients of Cluster2 might benefit from ICB treatment. Furthermore, we investigated the expression levels of several immune checkpoints aforementioned among two subtypes, and we found most of them were higher expressed in Cluster2, which indicated that patients of Cluster2 would benefit more from immune checkpoint inhibitors than those of Cluster1. Consistent with our hypotheses, patients in Cluster2 were demonstrated to be sensitive to anti-PD-1 therapy using SubMap analysis. Monoclonal antibodies targeting *PD-1* had shown positive outcomes in several human cancer types, including melanoma, renal cell carcinoma, and non-small cell lung cancer (64). Newly published studies provided evidence that *PD-1* blockade augmented antitumor immune response, chemokine transcripts expression, and T lymphocyte infiltration in GBM (65, 66). Although many clinical trials have demonstrated that the efficacy of ICB therapy for GBM remains unsatisfactory to date, Cloughesy et al. reported that the median overall survival for GBM patients received neoadjuvant (presurgical) *PD-1* blockade was much longer than the adjuvant group (417 days vs. 228.5 days, HR = 0.39, P < 0.05) (66). This discrepancy may result from the dampened immune responses and the suppression of cellular immunity caused by surgery itself. GBM patients of cluster2 in our study were proved to be more sensitive to anti-PD-1 treatment, so we have the reason to speculate that these patients may benefit from the neoadjuvant administration before surgery. In clinical practice, precision identification of patients of this GBM subtype with biopsy and then followed with anti-PD-1 treatment before tumor resection may significantly prolong the survival time for patients. Currently, several clinical trials are still undergoing to utilize anti-PD-1 for the treatment of GBM (NCT02311920 and NCT03726515) (67, 68).

Multiple factors were reported to affect the effectiveness of ICB treatment, such as copy number alterations (CNAs), tumor mutation burden, mutational signature, and T-cell signature (34). The source article of the melanoma transcriptome mainly discussed the role of the CNAs in modulating the response to CTLA-4 and PD-1 blockade. However, to our best knowledge, none developed tool or algorithm is available for comparing the similarity of the mutational or CNA data. The gene expression profile contains rich information on the biological processes, including immune response. The high similarity of the gene expression patterns indicates the similarity of the genome to some extent and then may reflect the similar responses to the ICB therapy. Several studies have utilized the gene expression data of various types of cancer receiving ICB therapy to predict the possible responses to immunotherapy in the interested cancer types. For example, Ock et al. developed a transcriptional predictor of immunotherapy response for pan-cancer using publicly available data for the ICB therapy with gene expression profiles in several cancer types (69). Jiang et al. developed a computational method based on gene profiling data in several tumor types for anti-PD-1 and anti-CTLA4 therapies to accurately predict the outcome of melanoma patients treated with ICB (70). Zeng et al. included several genomic and transcriptomic datasets from patients with different cancer types treated with immunotherapy in their study to evaluate the efficiency of the tumor microenvironment score they developed in predicting the immunotherapeutic benefits (71). Among these studies, the gene expression data from patients with different tumors treated with ICB therapies, including renal cell carcinoma, melanoma, non-small cell lung carcinoma, head and neck squamous cell carcinoma, were applied not limited to the same cancer types. Thus, we have reasons to shift our gaze to the transcriptomic data for comparing the similarity of the gene profiles of the two clusters we identified with the data of other cancer types received ICB therapies.

We focused on the AS events-based risk model again and intended to explore whether this AS events signature is closely associated with the tumor microenvironment. Apart from neoplastic and immune cells, subpopulations of stromal cells also exist in the complex tumor niche, such as endothelial cells, fibroblasts, reactive astrocytes, and microglial cells, and feedbacks from these cell subsets can driver malignant progression (47, 72). The negative correlation between risk score and tumor purity indicated that AS events-based signature might influence the constituents of non-tumor cells in GBM samples. Meanwhile, the risk score was positively correlated to several gene sets of glioma-related immunosuppressive factors, suggesting that the worse prognosis of patients in the high-risk group may result from the enhanced immunosuppressive environment. A mass of genes known to be associated with the immunosuppressive function was generated to investigate how AS events-based signature functions as a regulator of immunosuppression. Large numbers of immunosuppressive genes were positively correlated with the increasing risk score: *TIMP1* promoting recruitment of neutrophils to the liver to trigger the formation of the premetastatic microenvironment (73), *BIRC3* producing cytokines and chemokines by regulating nucleotide-binding and oligomerization signaling pathways (74), *ICAM1* boosting oral cancer progression by inducing CD163-positive macrophages adhesion (75), *CCL2* highly expressed in pancreatic tumors promoting anti-tumor immunity by increasing the infiltration of immunosuppressive CCR2+ macrophages (76), *LGALS1* inhibiting immunosuppressive cytokines by decreasing M2 macrophages and myeloid-derived suppressor cells in GBM (77), FOXP3+ Treg cells resulting in poor prognosis in various human cancers (78–80).

Splicing factors are known to function as oncogenes or tumor suppressors by regulating specific splice variants in glioma (81–83). Thus, we developed a correlation network between SFs and AS events to clarify the splicing regulatory mechanisms in GBM. Several factors have been reported essential to drive tumorigenesis among these crucial SFs, such as *TIA1* (84), *PQBP1* (85), *HSPB1* (86), and *EEF1A1* (87). Considering the expression levels of protein-coding genes were susceptible to the promoter loci methylation and copy number variation status, we investigated the influence of these kinds of factors on SFs expression. Therefore, we constructed a comprehensive network to help understand the potential regulatory pathways in cancer as well as motivate the development of novel target drugs for clinical treatment.

Although our research sheds new light on the novel molecular classification and immune microenvironment, we still have to acknowledge some limitations in the study. Firstly, our study was designed retrospectively, and findings should be further validated by prospective research. The prognostic value of AS events-based signature should be evaluated in clinical management. Secondly, the findings of the aberrant AS events and the construction of the developed prognostic model were merely based on the expression profiles of TCGA-GBM dataset, other independent GBM datasets should be included to further validate and expand these findings. We have searched large-scale gene expression profiles on GBM samples, but unfortunately only the TCGA project has the curated alternative splicing patterns of the corresponding tumor samples so that we can explore the potential value of the alternative splicing in GBM by integrating mRNA level information. Thirdly, limited by a scarcity of normal brain samples as references in the SpliceSeq database, specific AS events in GBM were not detected. We noticed that only five normal brain samples with both AS and gene expression profiles were included in the TCGA-GBM dataset. The huge difference in the size of the normal and GBM samples make it inapplicable for the identification of differentially expressed AS events. Comparing the differences of the AS patterns directly will inevitably introduce statistical bias, and the result may be unstable and incredible. Furthermore, our study was based on single-omics (AS events), so that the distinct molecular and clinicopathologic features among patients of high- and low-risk groups, as well as different subtypes, may result from intrinsic discrepancies of other factors, such as somatic mutation and DNA methylation. Also, we acknowledge that the RNA-seq data of GBM in the TCGA database was based on the bulk tumor sequencing, and the gene expression profiles cannot precisely represent the expression patterns of the cancer cells inside due to the inherent heterogeneity. The detailed characterization of the expression patterns of specific cancer cells needs the support of the single-cell sequencing method. Although several some computational approaches have been developed to estimate specific gene expression profiles for tumor cells inside the bulk samples, such as ISOpure and DeMixT, the prediction accuracy of these algorithms has not been validated in the TCGA-GBM cohort (88, 89). Applying these estimation methods to the unverified TCGA-GBM dataset directly may introduce unexpected bias. In addition, it is insufficient to predict the responses to immunotherapies for GBM merely based on AS events. Other omics data should be aggregated to develop a robust biomarker for immunotherapy response prediction.

In summary, our study identified significant prognosis related value of AS events in GBM and built an effective risk model to predict the survival outcomes for patients. Also, the aberrant alternative spliced variants were closely associated with the regulation of the immune microenvironment during the development of malignant tumors. The potential mechanisms that prognostic splicing factors affecting the overall survival of patients by regulating AS events were further exploited. Moreover, newly developed GBM classification based on AS events clustering analysis uncovered the inherent relevance of molecular and immune features. Therefore, this deep-mining analysis of AS patterns may provide some new perspectives to develop novel therapeutic strategies against GBM.

## Data Availability Statement

Publicly available datasets were analyzed in this study. These data can be found here: TCGA (https://portal.gdc.cancer.gov/) and TCGA SpliceSeq database (https://bioinformatics.mdanderson.org/TCGASpliceSeq).

## Author Contributions

LZ and PZ designed the study. LZ, JZ, and ZL performed research. YW provided data collection and visualization. LZ and SX drafted the manuscript. All authors contributed to the article and approved the submitted version.

## Funding

This study was supported by the National Natural Science Foundation of China (Grant No. 81673210).

## Conflict of Interest

The authors declare that the research was conducted in the absence of any commercial or financial relationships that could be construed as a potential conflict of interest.
