# Supplementary material for: Comprehensive Characterization of Alternative mRNA Splicing Events in Glioblastoma: Implications for Prognosis, Molecular Subtypes, and Immune Microenvironment Remodeling
Source: Front Oncol. 2021 Jan 26;10:555632. doi: 10.3389/fonc.2020.555632 (PMC7870873; doi:10.3389/fonc.2020.555632)
Supplement: Supplementary file 1 [file DataSheet_1.zip › Supplementary_Figures.docx]

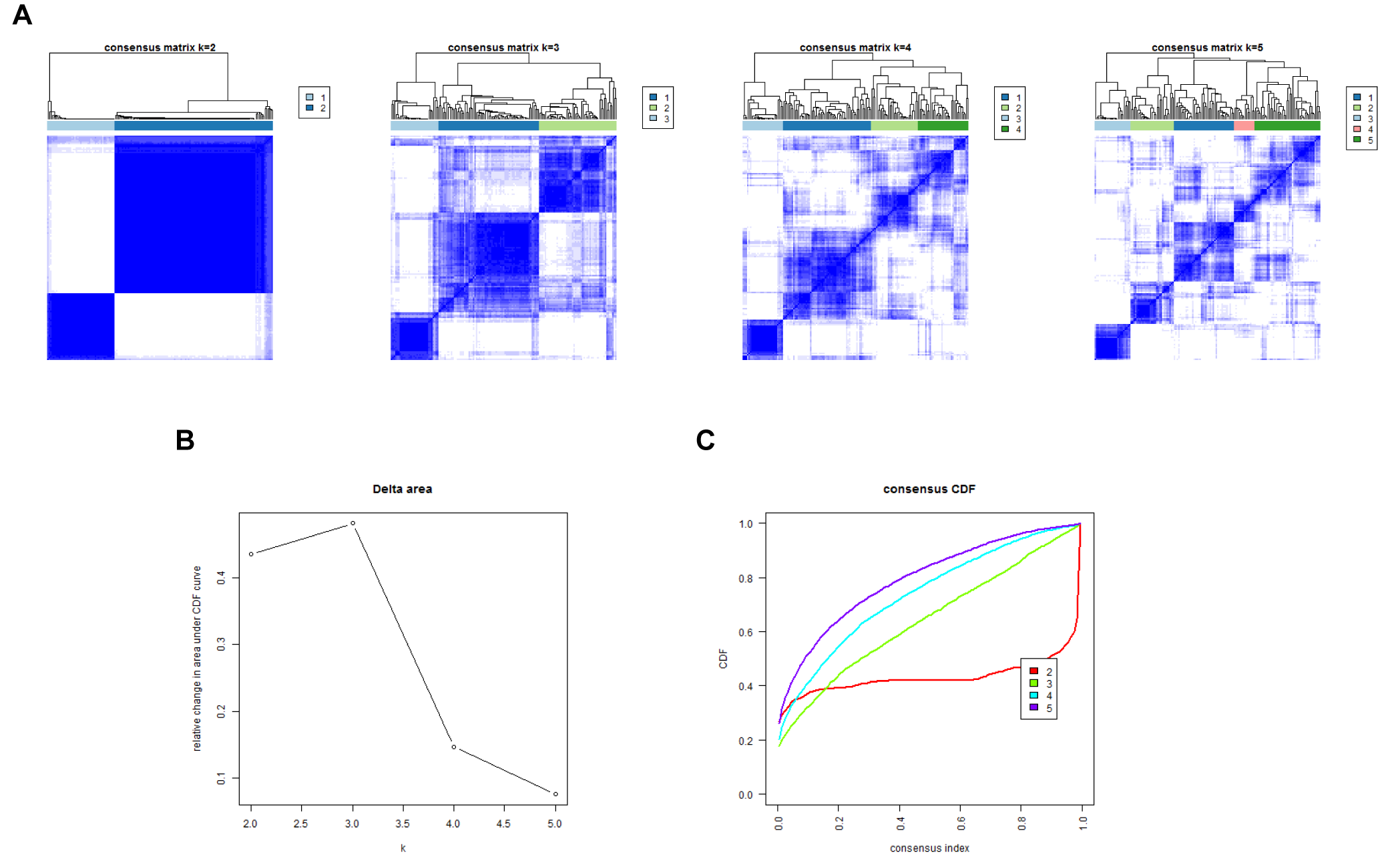


Supplementary Figure 1. Consensus clustering of GBM samples for k = 2 to k = 5. (A) The blue (consensus value = 0) and white (consensus value = 1) part of the heatmap indicate clustered and un-clustered samples, respectively. (B) Relative change in area under CDF curve for k = 2 to k = 5. (C) Consensus clustering CDF for k = 2 to k = 5.


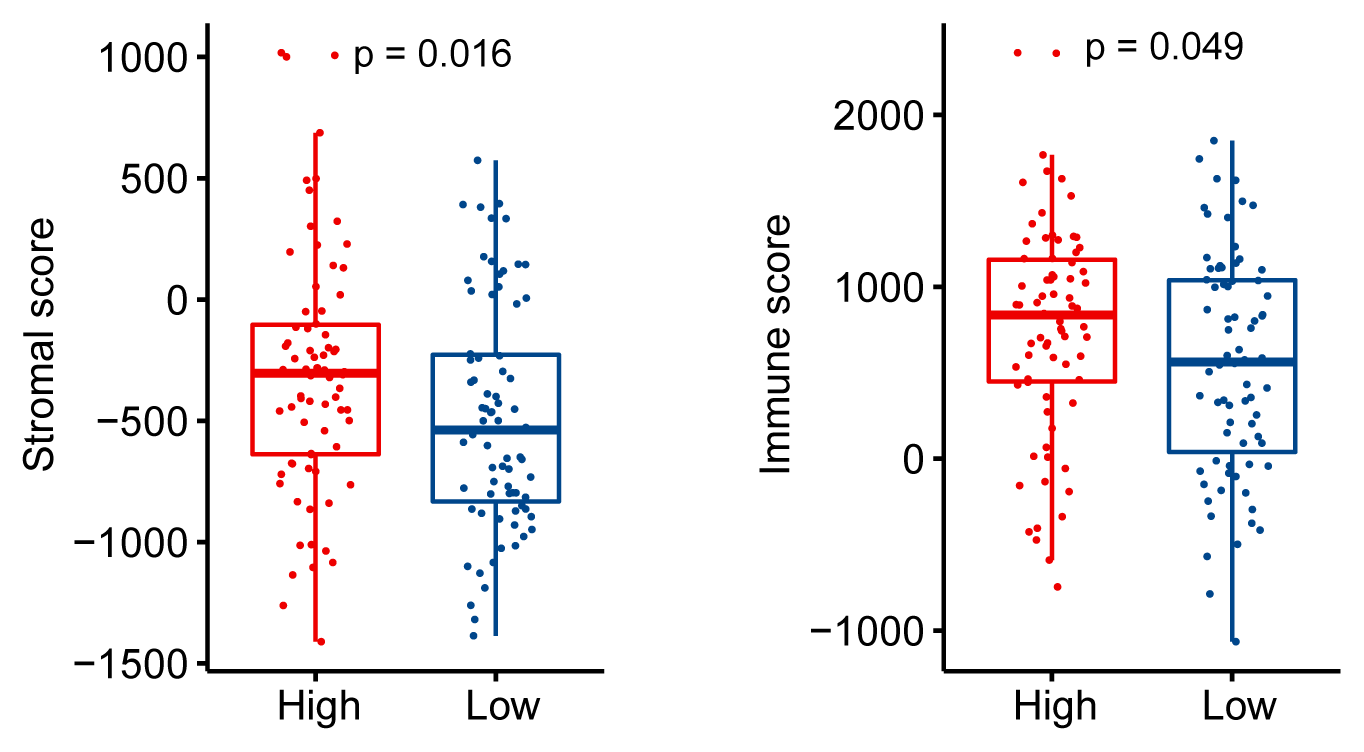


Supplementary Figure 2. Immune score and stromal score between high- and low-risk group patients. Score of each sample was obtained using the “ESTIMATE” R package.


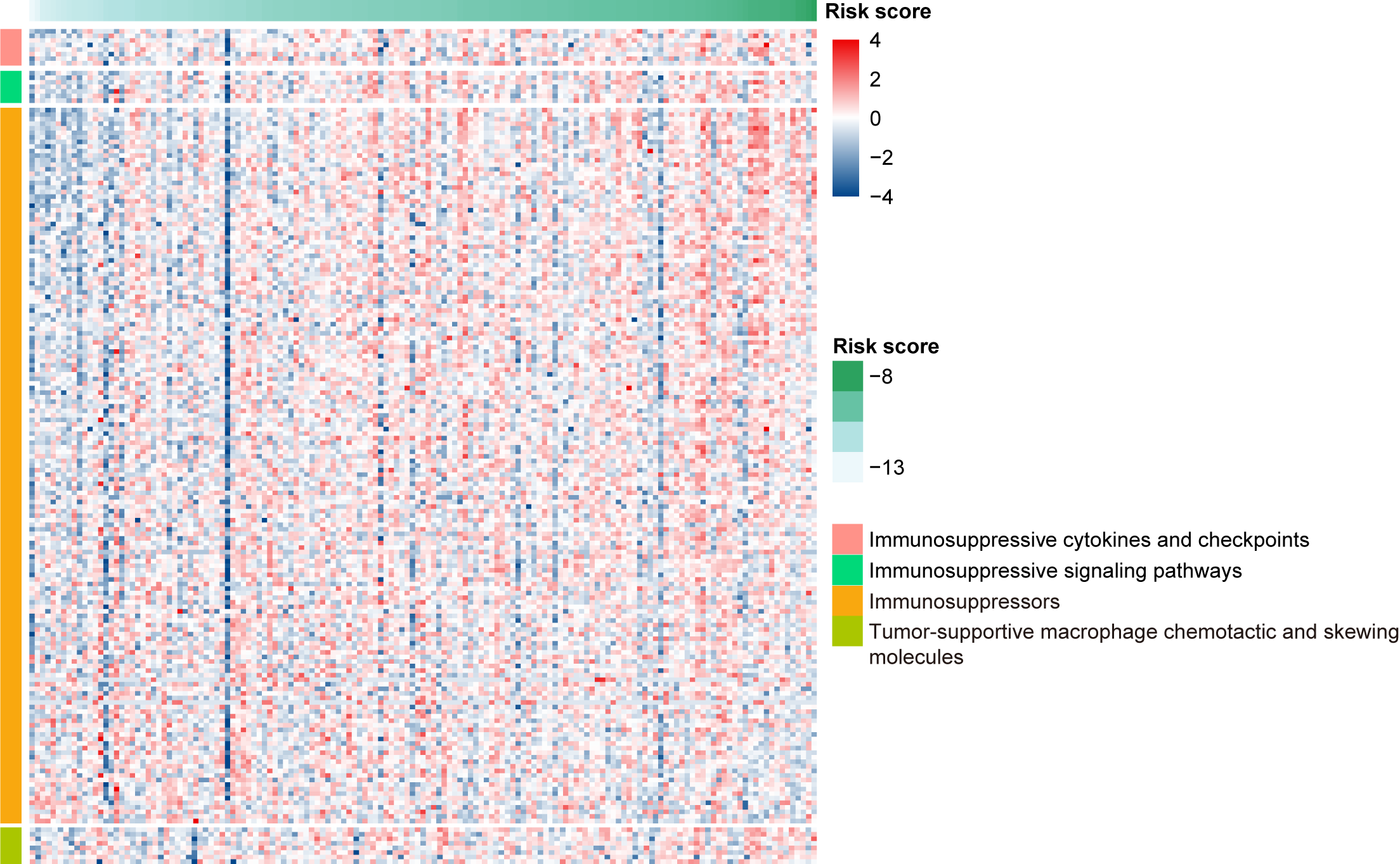


Supplementary Figure 3. Association between the risk score and immunosuppressive metagenes in GBM cohort.


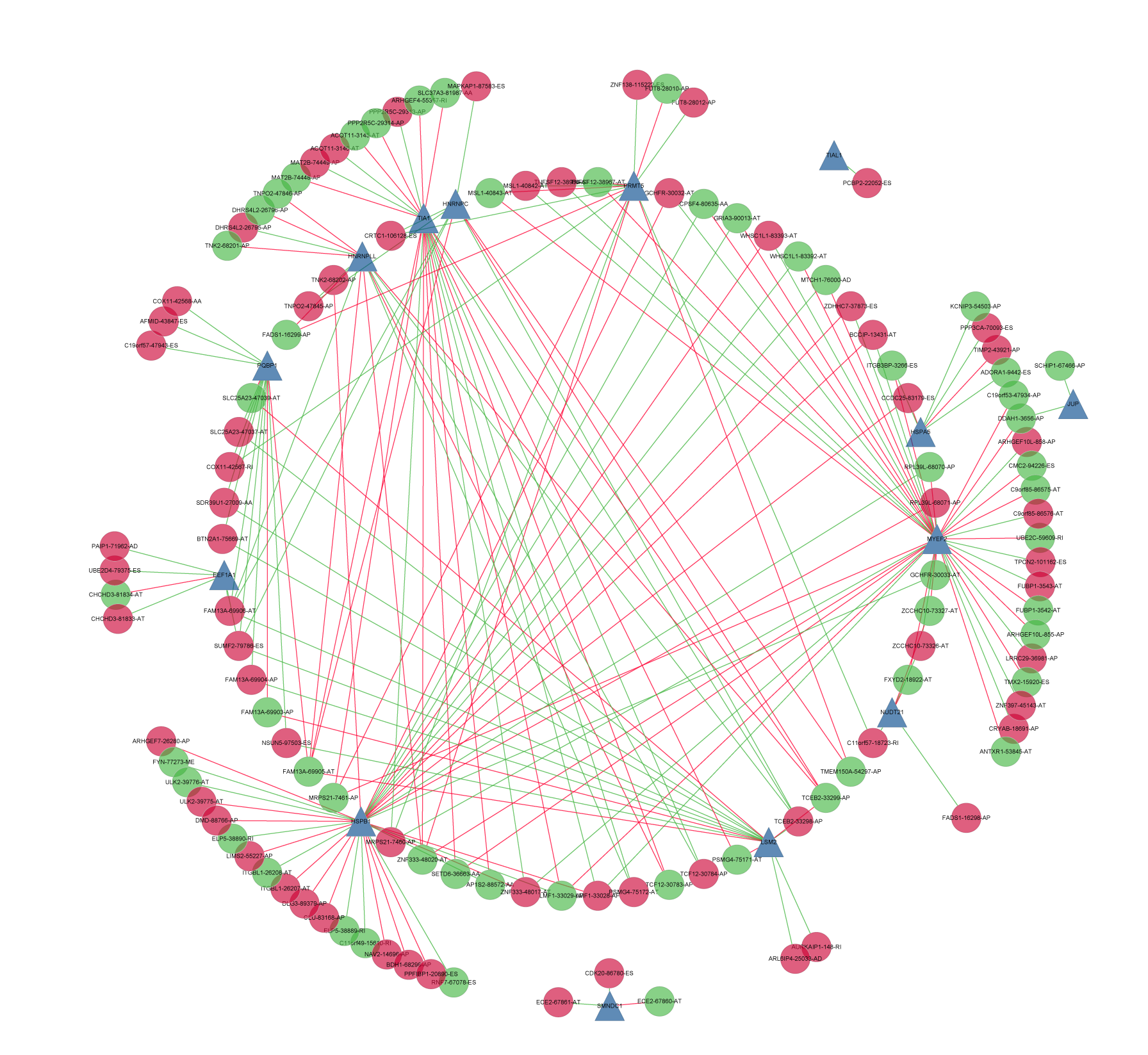


Supplementary Figure 4. Regulatory network between prognostic splicing factors (SFs) and survival-related alternative splicing (AS) events in GBM. Only significant correlations were plotted (|Pearson correlation coefficient| > 0.4 and P < 0.05). The red lines in the network indicate positive correlations between the expression of survival associated SFs and corresponding PSI values of AS events, while green lines indicate correlated negatively. SFs, favorable and poor prognosis AS events were plotted with triangles, green dots, and red dots, respectively.
