# Supplementary material for: Comprehensive Characterization of Alternative mRNA Splicing Events in Glioblastoma: Implications for Prognosis, Molecular Subtypes, and Immune Microenvironment Remodeling
Source: Front Oncol. 2021 Jan 26;10:555632. doi: 10.3389/fonc.2020.555632 (PMC7870873; doi:10.3389/fonc.2020.555632)
Supplement: Supplementary file 1 [file DataSheet_1.zip › Table_S3.docx]

Table S3. Univariate and multivariate analyses of clinical characteristics, IDH mutation status, MGMT methylation status, and AS-based risk score with overall survival in GBM cohort

| Variable | Univariate analysis | | Multivariate analysis | |
| --- | --- | --- | --- | --- |
|  | HR (95 % CI) | *P* | HR (95 % CI) | *P* |
| Age  >=60 *vs* <60 | 1.324 (0.919-1.906) | 0.131 | 1.422 (0.834-2.425) | 0.196 |
| Gender  Male *vs* female | 0.89 (0.608-1.301) | 0.547 |  |  |
| KPS  >=80 *vs* <80 | 0.697(0.431-1.127) | 0.141 | 0.91 (0.505-1.64) | 0.754 |
| *IDH* status  Wild-type *vs* mutation | 4.2 (1.683-10.481) | ***0.002*** | 0.831(0.17-4.047) | 0.818 |
| *MGMT* methylation  Unmethylated *vs* methylated | 1.843 (1.19-2.854) | ***0.006*** | 0.986 (0.538-1.808) | 0.964 |
| Risk score  High *vs* low | 2.718 (2.186-3.38) | ***<0.001*** | 2.496 (1.802-3.458) | ***<0.001*** |

Note: Bold italics indicate statistically significant values (P < 0.05).
